# Thermal Gelation for Synthesis of Surface-Modified Silica Aerogel Powders

**DOI:** 10.3390/gels7040242

**Published:** 2021-11-29

**Authors:** Kyoung-Jin Lee, Jae Min Lee, Ki Sun Nam, Haejin Hwang

**Affiliations:** Department of Materials Science and Engineering, Inha University, Incheon 22212, Korea; legna211@naver.com (K.-J.L.); 22201275@inha.edu (J.M.L.); skarltjs2@naver.com (K.S.N.)

**Keywords:** silica aerogel, thermal gelation, porous, thermal conductivity, hydrophobicity

## Abstract

A spherical silica aerogel powder with hydrophobic surfaces displaying a water contact angle of 147° was synthesized from a water glass-in-hexane emulsion through ambient pressure drying. Water glass droplets containing acetic acid and ethyl alcohol were stabilized in *n*-hexane with a surfactant. Gelation was performed by heating the droplets, followed by solvent exchange and surface modification using a hexamethyldisilazane (HMDS)/*n*-hexane solution. The pH of the silicic acid solution was crucial in obtaining a highly porous silica aerogel powder with a spherical morphology. The thermal conductivity, tapped density, pore volume, and BET surface area of the silica aerogel powder were 22.4 mW·m^−1^K^−1^, 0.07 g·cm^−3^, 4.64 cm^3^·g^−1^, and 989 m^2^·g^−1^, respectively. Fourier transform infrared (FT–IR) spectroscopy analysis showed that the silica granule surface was modified by Si-CH_3_ groups, producing a hydrophobic aerogel.

## 1. Introduction

A silica aerogel is a mesoporous solid with outstanding properties, including low thermal conductivity, a low dielectric constant, a low refractive index, and high specific surface area. Silica aerogels are considered as promising materials for thermal insulation [1,2], anti-reflection coatings [3], low dielectrics [4], supports for cosmetics [5], adsorbents [6,7,8], and viscosity agents [9].

Silica aerogels can be fabricated in the form of a monolith or a powder. Silica aerogel monoliths are normally produced by supercritical or ambient pressure drying of a wet gel derived via hydrolysis, and polymerization of alkoxide- or water glass-based precursor solutions [10]. Wei et al. reported an ambient pressure-dried silica aerogel monolith with multiple surface modifications, low thermal conductivity (36 mW/mK), and high porosity (97%) [11]. However, the repeated modification process to transfer hydrophilicity to the hydrophobic surface of the silica aerogel monolith is tedious and requires an extremely long processing time. The reactions between the surface modification agent and silanol groups (Si OH) are diffusion-limited processes, suggesting that the processing time for surface modification increases in proportion to the size of the silica aerogel sample. In addition, silica aerogel monoliths are fragile [12].

Silica aerogel powders and granules are easily and inexpensively fabricated and have a short processing time compared with silica aerogel monoliths. Researchers have recently reported novel methods for rapid synthesis of hydrophobic silica aerogel powders and granules using ambient pressure drying. Bhagat et al. proposed a one-step process with simultaneous surface modification, solvent exchange, and sodium ion removal [13]. Huber et al. presented a one-pot synthesis method for silica aerogel granulates [14]. They argued that gelation after surface modification is crucial for reducing the amount of solvent and production time. In our previous study focusing on catalysts for hydrolysis and condensation of water glass, we proposed a novel fast synthesis technique for spherical silica aerogel powders with a narrow particle size distribution. This synthesis technique reduced the total processing time to less than 2 h [15]. However, silica aerogel powders using the aforementioned fast synthesis processes exhibit some drawbacks. The powder quality is somewhat poor. Tapped density and pore volume are lower than for supercritically dried aerogels [10,11,16]. In addition, the silica aerogels lack particle size homogeneity.

Silica aerogel powders can be prepared by crushing bulk dried silica aerogel or using emulsion polymerization techniques. Although it appears simple and straightforward, crushing silica aerogel bulk is cumbersome, and the resulting powder is bulky. In addition, size and shape control is challenging. Emulsion polymerization is a promising technique for controlling the size and shape of silica aerogel particles. Spherical silica aerogel powder is produced from a water glass-in-hexane emulsion. The particle size of the silica aerogel can be determined by the water glass droplet size, which depends on the force applied to the homogenizer and the emulsifier content.

The silica aerogel powder produced by emulsion polymerization in our previous study had a tapped density of 0.12 g·cm^−3^, a pore volume of 2.35 cm^3^·g^−1^, and a thermal conductivity of 26 mW·m^−1^K^−1^, somewhat inferior to commercially available silica aerogel powders. We believe that the inferior properties are attributed to inhomogeneous hydrolysis and gelation in the water glass-in-hexane emulsion. In this study, a novel synthesis technique is proposed to produce high-quality spherical silica aerogel particles. We used a novel gelation process (thermal gelation), used for a sol-gel transition of natural polymers such as methylcellulose [17,18]. Water glass droplets containing acid and a gelation catalyst were stabilized in *n*-hexane with a surfactant, followed by thermal gelation, surface modification, and solvent exchange.

## 2. Materials and Methods

A water glass sodium silicate solution (silica content: 28–30 wt.%, SiO_2_:Na_2_O = 3.4:1, Young Il Chemical Co., Ltd., Incheon, Korea) was used as the starting material. Initially, the water glass solution was diluted to 5.3–8.7 wt.% with deionized water; 75 mL of water glass, 5 mL of acetic acid (99.5%, Samchun Pure Chemical, Pyeongtaek, Korea), and 5 mL of ethyl alcohol (95.0%, Samchun Pure Chemical) were mixed simultaneously. Ethyl alcohol was used as a condensation (gelation) catalyst, and as a con-solvent because a protic solvent such as ethyl alcohol can promote condensation. Next, 85 mL of *n*-hexane (95%, Samchun Pure Chemical) containing a surfactant, sorbitan monooleate (Span80, Junsei Chemical Co., Ltd., Tokyo, Japan), was added to the water glass/acetic acid/ethyl alcohol solution. The water glass solution to *n*-hexane ratio was fixed at 1. Water glass and *n*-hexane were emulsified using a homogenizer (UltraTurrax IKA T25:S25D-10G-KS, IKA Werke, Konigswinter, Germany) at 6000 rpm for 10 min. A stable water glass-in-hexane emulsion was obtained and heated at 60 °C for condensation (thermal gelation).

**Figure 1:**
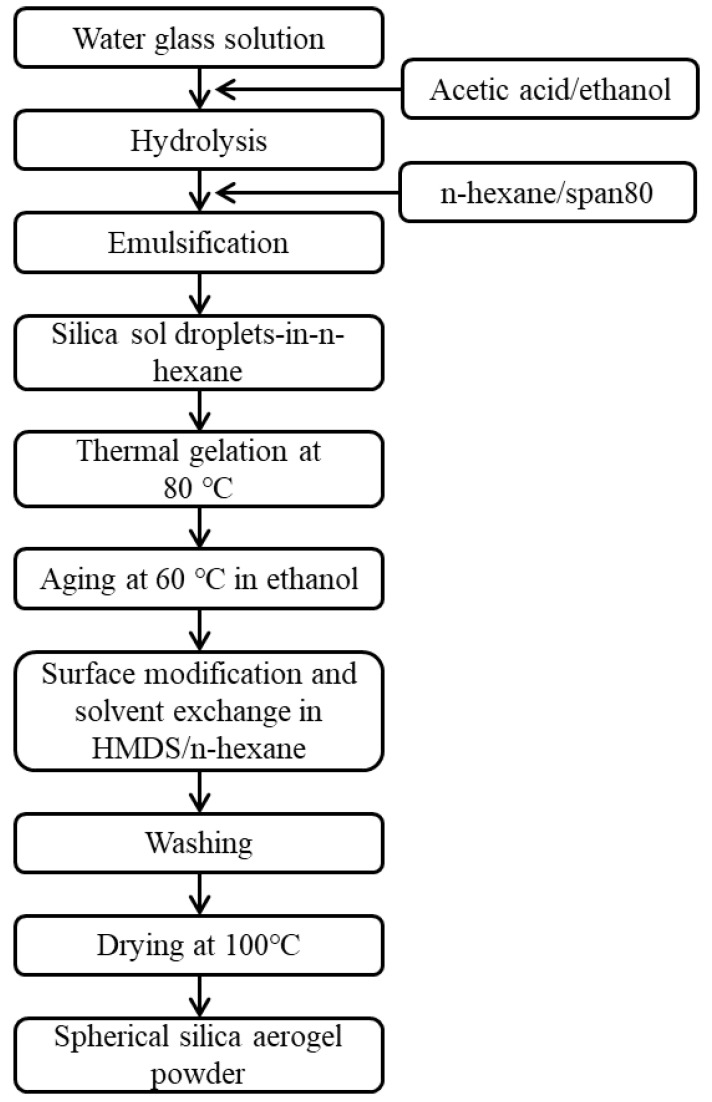
Experimental flow chart for synthesis of spherical silica aerogel powder.

Most of the *n*-hexane was drained from the emulsion, and the silica wet gel spheres were immersed in 150 mL of ethyl alcohol. Silica wet gel spheres were solvent-exchanged with ethyl alcohol, which can induce hydrogel-to-alcogel transformation. The surfaces of the silica alcogel spheres were chemically modified in 150 mL of 20% hexamethyldisilazane (HMDS, 98%, Samchun Pure Chemical)/*n*-hexane solution at 60 °C for 3 h. The silylated silica wet gel spheres were washed using an ethyl alcohol/*n*-hexane solution to remove the remaining surface modification agents and reaction products. The surface modification process was repeated three times. The silica wet gel spheres were dried at 100 °C in ambient pressure for 1 h. A schematic of the spherical silica aerogel powder preparation procedure is shown in Figure 1.

The tapped density of the aerogel powders was determined using a tapping density tester (TAP-2S, Logan Instruments Co., Somerset, NJ, USA). The viscosity of the silicic acid solution was measured using a viscometer (LVT B, Brookfield, Chander, AZ) at 25 °C. The surface area was determined by BET analysis from the amount of N_2_ gas adsorbed at different partial pressures (0.01 < p/p0 < 1, ASAP 2010; Micrometrics, Norcross, GA, USA). Fourier transform infrared (FT–IR) spectroscopy (FTS-165, Bio-Rad, Hercules, CA, USA) was used to confirm the surface chemical structure of the aerogels in the wavenumber range of 400–4000 cm^−1^. The contact angle of the water droplet on the silica aerogel powder was calculated from the height and width of the water droplet [19].

The thermal conductivity of the silica aerogel powder was measured using the heat flow metering method with a heat flow meter (HFM 436 Lambda, NETZSCH, Selb, Germany). A silica aerogel powder was placed between two flat plates (25 cm × 25 cm), with the upper and lower plates set at 35 °C and 15 °C, respectively. When thermal equilibrium was reached, the thermal conductivity was estimated using Fourier’s law. The thermal conductivity was calculated from the heat flux, the thickness of the silica aerogel powder, and the temperature gradient of the two plates. The microstructure of the silica aerogel powder was observed using field-emission scanning electron microscopy (FESEM, S-4200, Hitachi, Tokyo, Japan).

## 3. Results and Discussion

Both homogeneous gelation and surface modification are required to obtain high-quality hydrophobic silica aerogel powders (with low bulk density and high pore volume) [20]. Generally, hydrophobic silica aerogels are synthesized through gelation of silica sol or silicic acid solution and subsequent solvent exchange (SE) and surface modification (SM) using hydrophobizing agents. The SE and SM processes require a long processing time. To reduce the processing time, a one-pot process with simultaneous gelation, SE, and SM via the co-precursor method has been proposed [13, 14]. However, the one-pot process results in some reduced physical properties, including density and pore volume [21].

In this study, acetic acid and ethyl alcohol catalysts were used for the hydrolysis and condensation of the water glass solution. Generally, silica sol made with weak acids such as acetic acid is less stable than sol made with strong acids such as hydrochloric and nitric acids. In our previous study, it was deduced that acetic acid and isopropanol catalysts for hydrolysis and condensation of water glass were crucial for spherical silica aerogel powder synthesis with a narrow particle size distribution and a shorter production time. The pH of the silica sol made with acetic acid was 4.5–5.0; the silica sol exhibited adequate gelation time, neither long nor short, suggesting that silica sol catalyzed with acetic acid is stable.

When acetic acid and ethyl alcohol catalysts were added to the water glass solution, gelation did not occur in this step because the concentration of water glass was low (5–8%) and the silica sol made with acetic acid and ethyl alcohol was sufficiently stable at pH = 4.5–5.0. Gelation occurred when the silica sol in the *n*-hexane emulsion was heated to 60 °C. A silica wet gel with a spherical morphology was obtained. 

**Figure 2:**
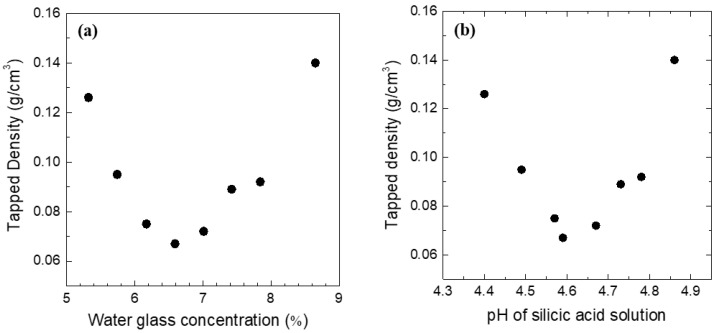
Tapped density of spherical silica aerogel powder as a function of water glass concentration (**a**) and pH of silicic acid solution (**b**).

Figure 2a,b show the tapped density of the obtained silica aerogel powders as a function of the water glass concentration and pH of the silicic acid solution. The lowest tapped density (0.067 g·cm^−3^) was obtained in a 6.6% water glass solution. Less than 6.6% water glass solution resulted in an increased tapped density. Above 6.6%, the increase in the tapped density was somewhat reasonable because the tapped density of the aerogel is proportional to the concentration of the starting water glass solution. In Figure 2a, below 6.6%, the tapped density increases again as a function of the water glass concentration. It can be inferred that this is associated with the pH of the silicic acid solution. The pH of the starting water glass solution was estimated to be 11.4 and does not depend on its concentration. In the silicic acid solution, a minimum tapped density was observed at pH = 4.5 (6.6% water glass solution).

Sarawade et al. addressed the mechanism of sol-gel polymerization from a water glass solution with an acetic acid catalyst. The gelation time decreased as the pH of the silicic acid solution increased; they showed that the minimum gelation time (5 min) was observed with a pH of 5–6 [22]. To obtain low-density silica aerogels, they used a silicic acid solution with a pH of 4. Above pH = 4, the gelation is too fast, leading to an extremely unstable silica sol. Below pH = 4, the hydrolysis and condensation reactions are not sufficient in the gelation process, which can affect the properties of the final silica aerogel products [23].

**Figure 3:**
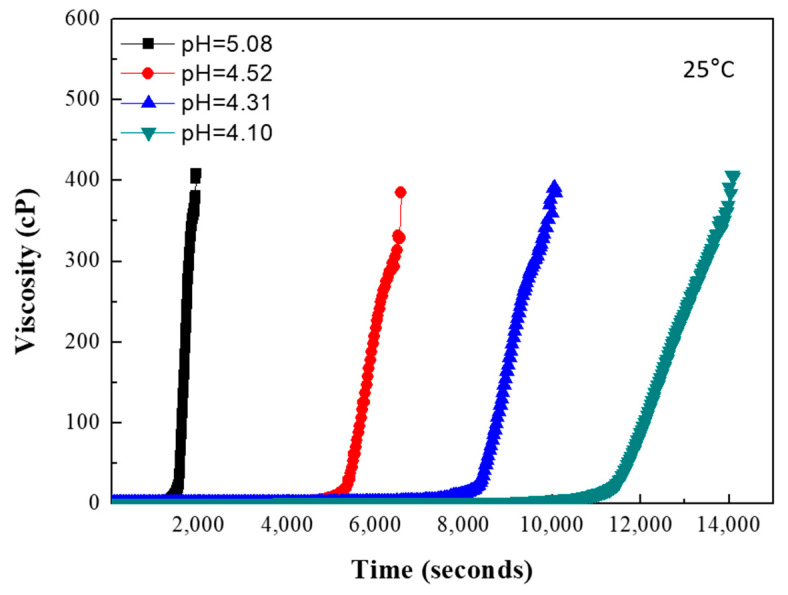
Viscosity of silicic acid solutions at different pH as a function of aging time.

Figure 3 shows the change in viscosity as a function of aging time for silicic acid solutions at different pH values. The viscosity abruptly increased to 400 cP from 30–180 min at pH = 5.08 and 4.10, respectively. The rate of viscosity change increased as the pH increased (acetic acid content decreased). Table 1 summarizes the gelation times of the silicic acid solutions and the tapped densities of the synthesized silica aerogel powders as a function of acetic acid content and pH. The concentration of the water glass solution was 6.6%. Gelation time is defined as the time after which the viscosity deviates from linearity [24]. The gelation time strongly depends on the pH of the silicic acid solution. As the acetic acid content increases, the pH value of the silicic acid solution decreases, and the gelation time gradually increases. The condensation rate is generally faster than the hydrolysis rate and increases with increasing pH from 4–10 [25].

From Table 1, the silica aerogel powder sample prepared with a water glass concentration of 6.6% and a pH value of 4.52 exhibited the lowest tapped density (0.090 g·cm^−3^), which was also observed in the aerogel powder sample with a water glass concentration higher than 6.6%. This result suggests that an optimum pH exists at which the aerogel powder sample exhibits the lowest tapped density. When the pH of the silicic acid solution is higher than approximately 4.5, gelation is too fast, which can lead to an increased tapped density owing to insufficient time for solvent exchange and surface modification. In Table 1, the tapped density of the silica aerogel powder sample prepared using a silicic acid solution with a pH of 5.08 was significantly higher than those of silica aerogel powder samples with pH values of 4.31 and 4.51. The tapped density increased at pH = 4.1 (acetic acid content: 10 mL) owing to the slow hydrolysis and condensation rate at a low pH. If the pH of the silicic acid solution is too low, hydrolysis, condensation, and surface modification reactions occur simultaneously, which can have a detrimental effect on the mesoporous structure development of silica aerogel.

**Figure 4:**
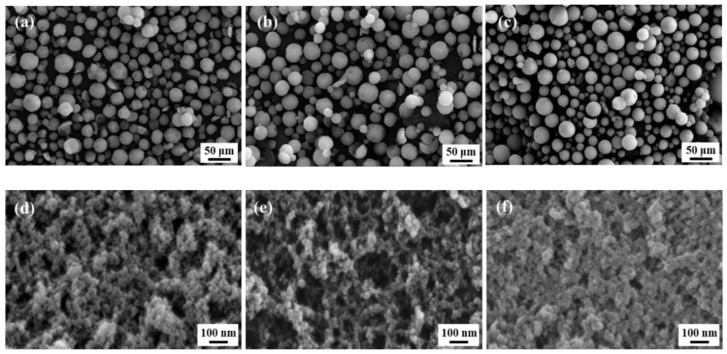
SEM images of silica aerogel powders prepared from (**a**,**d**) 5.7%; (**b**,**e**) 6.6%; (**c**,**f**) 8.7% water glass solutions.

Figure 4a–f show SEM images of silica aerogel powders prepared with different water glass concentrations. Figure 4a–c show that the obtained silica aerogel powders have a spherical shape, with average estimated sizes of approximately 10–30 μm, regardless of the water glass concentration (Figure S1). This result suggests that the water glass concentration does not significantly affect the size and morphology (sphericity) of the silica aerogel powders. High-magnification SEM images (Figure 4d–f) show that the silica particles possess a typical highly porous three-dimensional network structure consisting of silica nanoparticles, although their tapped densities are slightly different.

**Figure 5:**
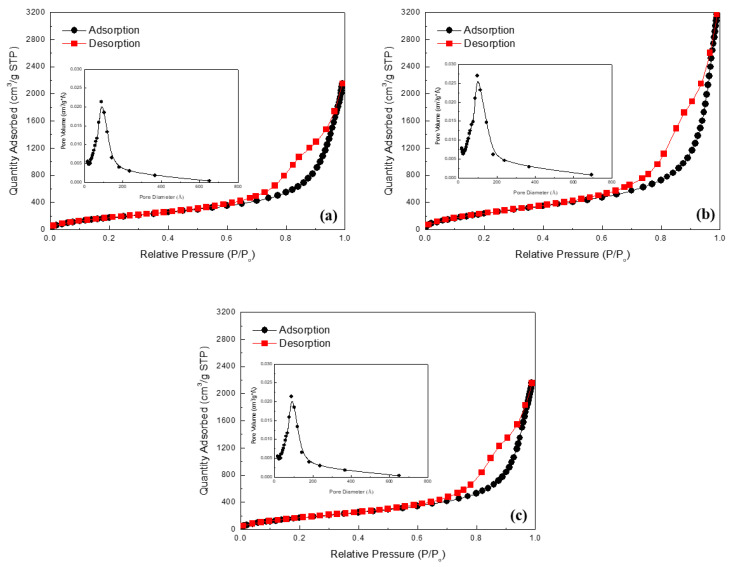
Adsorption–desorption isotherms of spherical silica aerogel powders synthesized from (**a**) 5.3%; (**b**) 6.6%; (**c**) 7.0% water glass solutions. Insets show pore size distribution based on BJH (adsorption).

N_2_ adsorption–desorption isotherms of the silica aerogel powder are shown in Figure 5. N_2_ absorption sharply increases near the high relative pressure area (Type IV adsorption–desorption isotherm with type H1 hysteresis loop), indicating that the silica aerogel is mesoporous [26,27]. It is known that hysteresis is attributed to capillary condensation and evaporation occurring in the mesopores [28]. The hysteresis loop in Figure 5b was found to be more significant, indicating many mesopores in the silica aerogel powder sample with a water glass concentration of 6.6%.

Table 2 summarizes the physical properties of silica aerogel powders prepared with different water glass concentrations. Tapped density, BET specific surface area, and pore volume strongly depend on the water glass concentration. However, the water glass concentration does not affect the size of the mesopores. The mean pore diameter ranges from 17–19 nm. However, in Figure 4, it appears that the macropore size and distribution changes with the water glass concentration. This observed phenomenon is associated with the difference in agglomeration behavior between secondary silica particles. Depending on the pH of silicic acid solution with different water glass concentrations, silica aerogels had different macrostructures. The agglomeration between silica particles increases with an increase in the pH of the silicic acid solution [29], leading to the formation of macropores, as can be seen in Figure 4b. In the case of 8.7% of water glass concentration, the silica aerogel possessed denser macrostructure than 5.7% and 6.6% aerogel samples, which is due to the large shrinkage during ambient pressure drying.

In Table 1, the silica aerogel powder prepared using a 6.6% water glass concentration exhibits the maximum BET specific surface area of 989 m^2^·g^−1^, and a pore volume of 4.64 cm^3^·g^−1^. The excellent properties of the 6.6% water glass solution sample are the result of a more porous microstructure and a slightly smaller particle size than the 5.7% and 8.7% samples. An adequate water glass concentration and pH are crucial for the silicic acid solution to obtain a silica aerogel powder with a low bulk density and a high pore volume.

**Figure 6:**
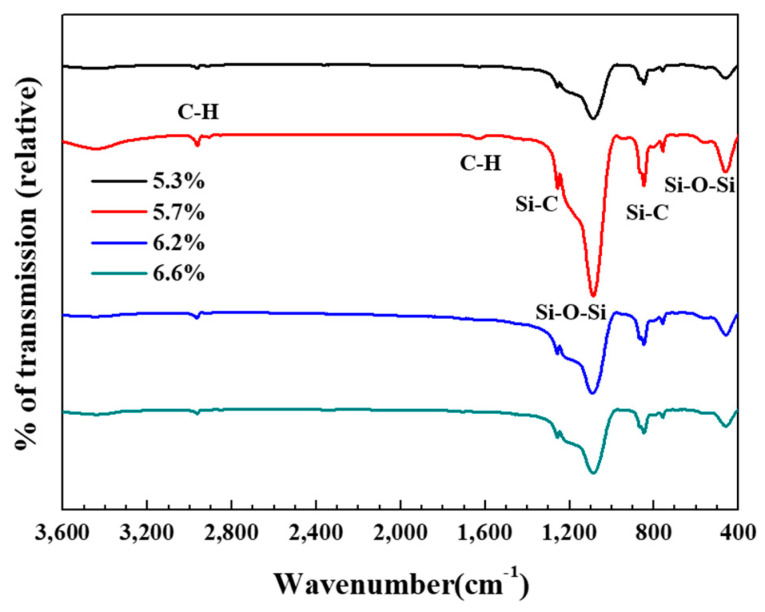
FT-IR spectra of silica aerogel powders prepared from water glass solutions with different concentrations.

**Figure 7:**
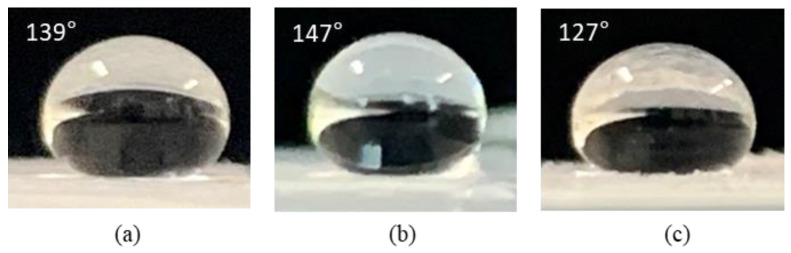
Photographs of water droplets on silica aerogel powders prepared with water glass solution concentrations of (**a**) 5.7%; (**b**) 6.6%; (**c**) 8.7%.

Fourier transform infrared (FTIR) spectroscopy was used to confirm the hydrophobicity of the silica aerogel powder samples; the corresponding FTIR spectra are shown in Figure 6. According to previous studies [30,31], the absorption peaks near 1090 cm^−1^, 760 cm^−1^, and 460 cm^−1^ can be attributed to the asymmetric, symmetric and bending modes of Si-O-Si, respectively. These peaks are characteristic of typical silica aerogel network structures. The peaks at 1260 cm^−1^ and 850 cm^−1^ indicate the presence of a Si-C bond; the peaks at 2960 cm^−1^ and 1600 cm^−1^ correspond to C-H stretching. Peaks corresponding to the stretching vibration of O-H (3455 cm^−1^ and 1635 cm^−1^) and Si-OH (~800 cm^−1^) were not observed in any of the silica aerogel powder samples [32]. Thus, it can be inferred that the silica aerogel was modified into a hydrophobic form by the surface methyl groups (-CH_3_). Photographs of water droplets on the silica aerogel powders prepared with water glass concentrations of 5.7%, 6.6%, and 8.7% are shown in Figure 7. The contact angles of the silica aerogel powder from the 6.6% water glass solution exhibited the highest contact angle of 147° (hydrophobicity); the contact angle decreased with increasing tapped density of the silica aerogel powder, suggesting that the contact angle (good hydrophobicity) is closely related to the tapped density of the silica aerogel powder. In general, the density of the silica aerogel depends on the degree of surface modification by functional groups. In other words, the high degree of modification results in low density because the pore water can be effectively exchanged by ethyl alcohol and *n*-hexane. Thus, it appears that the surface of the low-density silica aerogel is modified with large number of function groups, which can lead to good hydrophobicity.

The spherical silica aerogel powders prepared with water glass concentrations of 5.3% and 6.6% exhibited thermal conductivities of 24.2 mWm^−1^·K^−1^ and 22.4 mWm^−1^·K^−1^, respectively. Within our system, silica aerogel powder with a lower tapped density and higher pore volume exhibits a lower thermal conductivity. Although the thermal conductivity (22.4 mW·m^−1^·K^−1^) obtained in this study is somewhat lower than that of previously reported silica aerogel powder (~14 mW·m^−1^·K^−1^) [14], it is comparable to that of commercially available silica aerogel powder with a similar particle size [33]. Thus, the silica aerogel powder obtained in this study can be used commercially in the field of superinsulation.

## 4. Conclusions

Silica aerogel powders with spherical morphology and hydrophobic surfaces displaying a water contact angle of 147° can be synthesized from water glass solutions via emulsion polymerization, thermal gelation, and ambient pressure drying. In this study, acetic acid and ethyl alcohol were used as hydrolysis and condensation catalysts, respectively. The ratio of acetic acid content to water glass concentration, affecting the pH value of the silicic acid solution, was crucial in obtaining a highly porous spherical silica aerogel powder. The silica aerogel powder obtained from a silicic acid solution with a pH of approximately 4.5 exhibited the lowest thermal conductivity, and the greatest specific surface area and pore volume. As the acetic acid content was increased (as the pH of the silicic acid solution decreased), the gelation time was significantly increased, which led to an increased tapped density. If the pH of the silicic acid solution was above 4.5, the gelation rate was too fast and difficult to control, and physical properties of the silica aerogel powder such as pore volume and specific surface area deteriorated significantly.

## Figures and Tables

**Table 1:** Gelation time of silicic acid solutions with different pH and tapped densities of silica aerogel powders (concentration of water glass solution: 6.6%).

Acetic Acid Content (mL)	pH of Silicic Acid Solution	Gelation Time (min)	Tapped Density (g·cm^−3^)
3	5.08	24	0.227
5	4.52	85	0.090
7	4.31	128	0.097
10	4.10	180	0.236

**Table 2:** Physical properties of silica aerogel powders synthesized from different water glass concentrations.

Water Glass Concentration (%)	Specific Surface Area (m^2^·g^−1^)	Pore Volume (cm^3^·g^−1^)	Mean Pore Diameter (nm)
5.3	719	3.11	17.3
5.7	731	3.20	17.5
6.2	680	3.20	18.8
6.6	989	4.64	18.8
7.0	709	3.14	17.7

## Data Availability

Not applicable.

## Supplementary Materials

The following are available online at https://www.mdpi.com/article/10.3390/gels7040242/s1, Figure S1: Particle size distribu-tions of silica aerogel powder samples prepared with different water glass concentrations.
