# Supplementary material for: Thermal Gelation for Synthesis of Surface-Modified Silica Aerogel Powders
Source: Gels. 2021 Nov 29;7(4):242. doi: 10.3390/gels7040242 (PMC8701169; doi:10.3390/gels7040242)
Supplement: Supplementary file 1 [file gels-07-00242-s001.zip › gels-1465652-supplementary.pdf]

Supplementary materials

Thermal Gelation for Synthesis of Surface-Modified Silica Aerogel Powders

Kyoung-Jin Lee, Jae Min Lee, Ki Sun Nam and Haejin Hwang *

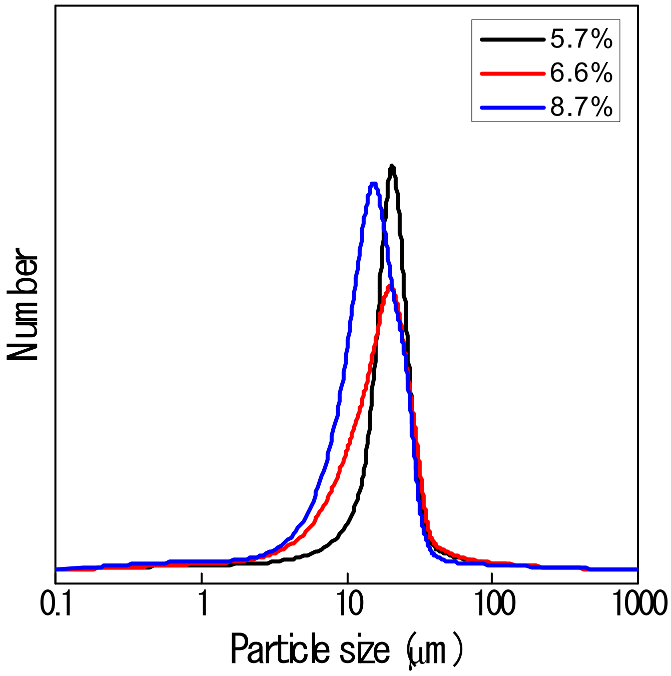

Figure S1. Particle size distributions of silica aerogel powder samples prepared with different water glass concentrations.
